# Forty Years of Erratic Insecticide Resistance Evolution in the Mosquito Culex pipiens

**DOI:** 10.1371/journal.pgen.0030205

**Published:** 2007-11-16

**Authors:** Pierrick Labbé, Claire Berticat, Arnaud Berthomieu, Sandra Unal, Clothilde Bernard, Mylène Weill, Thomas Lenormand

**Affiliations:** 1 Equipe Génétique de l'Adaptation, Institut des Sciences de l'Evolution, CNRS Université Montpellier 2, Montpellier, France; 2 Centre d'Ecologie Fonctionnelle et Evolutive, UMR 5175, Montpellier, France; Princeton University, United States of America

## Abstract

One view of adaptation is that it proceeds by the slow and steady accumulation of beneficial mutations with small effects. It is difficult to test this model, since in most cases the genetic basis of adaptation can only be studied a posteriori with traits that have evolved for a long period of time through an unknown sequence of steps. In this paper, we show how *ace-1*, a gene involved in resistance to organophosphorous insecticide in the mosquito Culex pipiens, has evolved during 40 years of an insecticide control program. Initially, a major resistance allele with strong deleterious side effects spread through the population. Later, a duplication combining a susceptible and a resistance *ace-1* allele began to spread but did not replace the original resistance allele, as it is sublethal when homozygous. Last, a second duplication, (also sublethal when homozygous) began to spread because heterozygotes for the two duplications do not exhibit deleterious pleiotropic effects. Double overdominance now maintains these four alleles across treated and nontreated areas. Thus, *ace-1* evolution does not proceed via the steady accumulation of beneficial mutations. Instead, resistance evolution has been an erratic combination of mutation, positive selection, and the rearrangement of existing variation leading to complex genetic architecture.

## Author Summary

Adaptation is not always a straightforward process, and often results from natural selection tinkering with available variation. We present in this study just such a tortuous natural selection pathway, which allows the mosquito Culex pipiens to resist organophosphorous insecticides. In the Montpellier area, following the use of insecticide to control mosquito populations, a high-resistance allele of the insecticide target enzyme appeared. But this allele also displayed strong deleterious side effects. Recently, several duplicated haplotypes began to spread in natural population that put in tandem a susceptible and a resistant allele. We show that the duplicated haplotypes actually display reduced side effects compared to the resistant allele when in the heterozygous state, but also new and strong costs in the homozygote. This pattern leads to an unexpected equilibrium between four different alleles across treated and nontreated areas. The story of resistance in C. pipiens is indeed far from a slow progression toward a “perfect” adaptation. Rather, selection for resistance to insecticide is a long process of trial and error leading to an uncommon genetic architecture.

## Introduction

Adaptation is often envisioned as a slow and regular improvement, a view embodied by Fisher's geometrical model of adaptation, whereby mutations fix if they bring the current phenotype closer to an optimum [1,2]. However, the trajectory towards the optimum can take a truly tortuous path [3], as adaptation uses almost any allele that brings it closer to the optimum, regardless of its negative side effects. “Evolution is a tinkerer,” as emphasized by Jacob [4], may indeed be more than a pretty metaphor. That beneficial mutations often have deleterious pleiotropic effects is well established (i.e., they generate a fitness cost [1,5–8]). Pleiotropy causes an “evolutionary inertia” [9] whereby beneficial mutations often only ameliorate the side effects of the last beneficial mutation. This process of “amelioration” [10] can follow a scenario a la Fisher [11] with modifiers or compensatory mutations occurring at different loci, or with allele replacement at the same locus (Haldane [12]). Both cases have been reported (e.g., [10,13–16]). However, it is perhaps less often appreciated that pleiotropy can have more dramatic consequences. First, the pleiotropic effects of beneficial mutations may be more complex than simply deleterious. Second, they may trigger the evolution of the genetic architecture and gene number. This paper illustrates these two aspects with the tortuous path taken during the evolution of insecticide resistance in Culex pipiens mosquitoes in southern France.

In natural populations of the mosquito Culex pipiens, various resistance genes have been selected over the course of ∼40 y of control using organophosphorous (OP) insecticides (see [17] for a review). OP insecticides kill by inhibiting acetylcholinesterase (AChE1) in the central nervous system. The genetic basis of OP resistance involves two main loci, the super-locus *Ester* and the locus *ace-1*, both of which have major resistance alleles. Strong direct and indirect evidence indicate that the resistance alleles have a “fitness cost”; i.e., they are selected against in the nontreated areas (see reviews in [17,18]). At the *Ester* locus, the different resistance alleles are not identical and one has slowly replaced the other [15,16].

At the *ace-1* locus, the resistance allele present worldwide, *ace-1^R^*, displays a single amino acid mutation (G119S), changing the glycine at position 119 into a serine. This large effect point mutation confers high resistance towards OP insecticides due to lower affinity and has arisen independently in several mosquito species [19–21]. However, this mutation also exhibits a strong deleterious side effect: G119S is less efficient at degrading acetylcholine (ACh) than the susceptible variant of AChE1. Overall, G119S causes a more than 60% reduction in enzymatic activity in the absence of insecticide [22], which alters the optimal functioning of cholinergic synapses of the central nervous system and probably causes the various developmental and behavioral problems that have been identified in individuals carrying *ace-1^R^* [23–25]. Field surveys also quantified the overall fitness cost of this allele in the wild. Fitness values are 1 and 0.89 for a susceptible and resistant homozygote, respectively, in the absence of insecticide (i.e., when only the cost applies [26,27]). However, in a treated habitat, only *ace-1^R^*-carrying individuals survive.

A few years after the appearance and spread of *ace-1^R^*, several duplications of the *ace-1* gene appeared in wild populations, each involving a resistant and a susceptible copy of the *ace-1* gene on the same chromosome [28–30]. We refer to these new duplicated haplotypes as *ace-1^D^*. The *ace-1* copies involved in these duplicated haplotypes are barely distinguishable from local single-copy alleles. In particular, within a population, the sequence of the local *ace-1^D^* resistant copy is always identical to the sequence of the single-copy *ace-1^R^* allele [30]. In the Montpellier area of Southern France, the appearance of a duplicated haplotype was indirectly inferred and traced back to at least 1993 [29]. More recently, sequence data of *ace-1* confirmed the presence of duplication, but surprisingly also revealed the presence of two distinct duplicated haplotypes in Montpellier area (*ace-1^D2^* and *ace-1^D3^*) [30]. Other independent duplications were also found in the Caribbean (Martinique, *ace-1^D1^*) and Philippines (Palawan, *ace-1^D4^*) [30].

Gene duplication is an important type of mutation because it fosters the evolution of new functions [12,31–33]. In the case of *ace-1*, a strong genetic constraint drives resistance evolution, as the degree of resistance and the ability to degrade ACh trade off; OP and ACh molecules compete for the same active site of AChE1. Duplication may be a way to disentangle the two functions, i.e., by improving synapse signalling and mosquito's fitness while maintaining resistance.

Thus, our working hypothesis is that the spread of *ace-1* duplications is driven by the increased AChE1 activity in individuals carrying *ace-1^D^* compared to those carrying *ace-1^R^*. In the last survey eight y ago [29], indirect estimation indicated a rapid initial increase of *ace-1^D^*, suggesting a strong selective advantage of *ace-1^D^* over *ace-1^R^* (in the range of 3%–6% of *ace-1^R^* fitness [29]). Based on these previous findings, we expected the duplicated haplotype to quickly replace *ace-1^R^*. The aim of this study was to test this prediction and to understand more precisely the fitness relationship between the various genotypes (single resistance or susceptible alleles, duplicated haplotypes and their heterozygotes) in treated and nontreated areas. Surprisingly, we show that *ace-1^D^* did not replace *ace-1^R^*. Several experiments indicated that duplications, while favored when heterozygous, exhibit very strong deleterious effects when homozygous. Our work thus highlights a novel scenario of adaptive evolution and trade-offs that hinge on the genetic architecture underlying the expression of resistance variation.

## Results

In the following, and according to the nomenclature used in Labbé et al [30], the duplicated haplotypes *ace-1^D2^* and *ace-1^D3^* will be denoted D_2_ and D_3_, susceptible copies being denoted D_2_(S) and D_3_(S), and resistant copies, D_2_(R) and D_3_(R), respectively. D refers to either D_2_ or D_3_. The single copy resistant allele (*ace-1^R^*) will be denoted as R. Finally, single susceptible alleles (*ace-1^S^*) will be denoted S. The only S allele present in the strains used in laboratory experiments and originating from the susceptible reference strain SLAB [34] will be denoted S_SLAB_.

**Figure 1:**
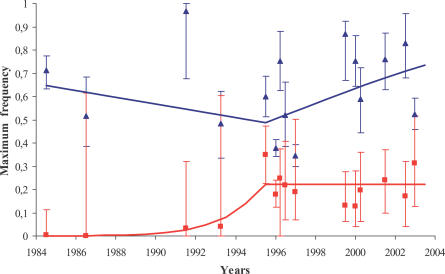
Time Variation of the Different Alleles' Maximum Frequency The evolution of the maximum frequency (coastal frequency) for each *ace-1* resistance allele estimated from the descriptive model is presented. The blue triangles and red squares represent, respectively, the maximum frequency of R and D (= D_2_ + D_3_) estimated for each cline independently (blue and red bars represent the confidence intervals, respectively), the blue and red lines representing the frequency estimated from the complete dataset conjointly (see text). Note that no tools are currently available to distinguish D_2_ and D_3_ in the field, only their total frequency being thus estimated (see Materials and Methods).

We first analyzed the frequency variation of R and D (= D_2_ + D_3_) across treated and nontreated areas around Montpellier from 1986 to 2002, using a purely descriptive model (see Methods). As in previous analyses [26,27,29], we found that their frequency showed clinal variation: R and D are more frequent in the treated than the nontreated area (Table 1). The straightforward interpretation of these clines is that R and, to a lesser extent, D have a selective advantage in the presence of insecticide over S, but that they have a fitness cost in its absence. Migration leads to the smooth decline of frequency in the nontreated area and prevents fixation of resistance in the treated area. We also confirm that D was rare until around 1993, when it started to rapidly increase in frequency. Surprisingly, D frequency stopped increasing around 1996 and reached a plateau at about 22% (this holds when only summers clines are considered, analysis not shown) (Figure 1; Table 2);.

**Table 1:**
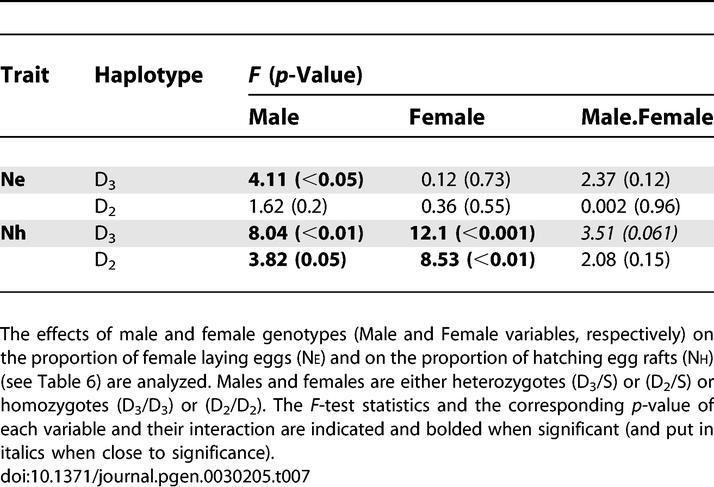
Parameters of the Best-Fitted Clines

**Table 2:**
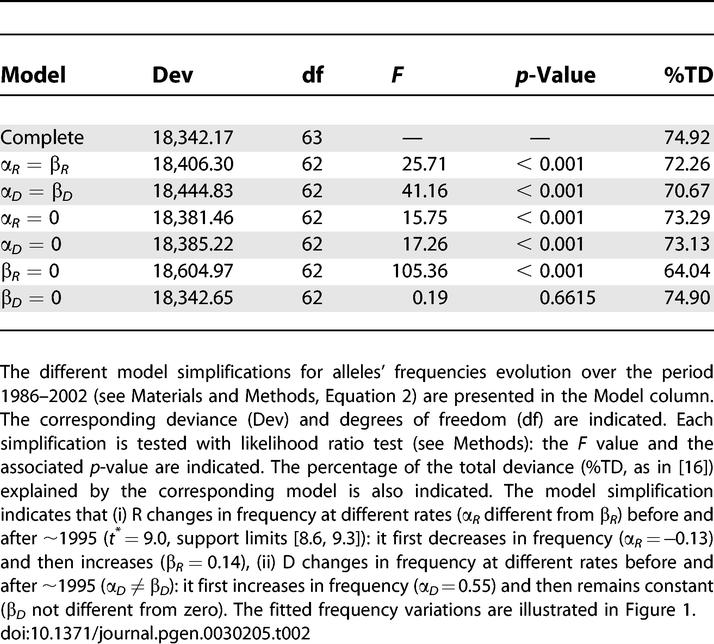
Time Variation of Resistance Clines

**Figure 2:**
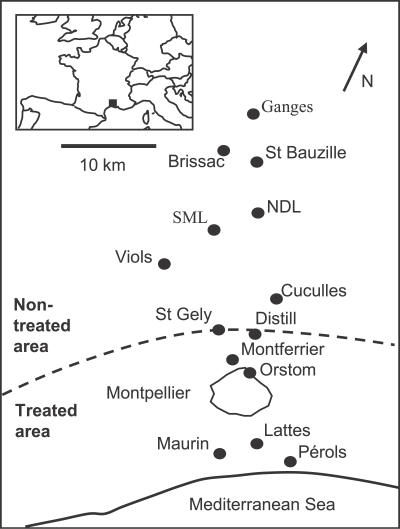
Sample Site Locations in the Northwest Southeast Transect in the Montpellier Area Samples are indicated with black circles. The dashed line represents approximately the border between treated and untreated areas (modified from [16]). C. pipiens is present in the whole area.

However, this analysis does not distinguish D_2_ and D_3_ duplications. To assess the frequency and the distribution of D_2_ and D_3_ along the cline, five populations were sampled in 2005, on the same transect as for cline analysis, two in the treated area (Maurin and Lattes), one at the limit between the two areas (Distill), and two in the nontreated area (Viols and Ganges, Figure 2). Molecular tests available allow detection of D_2_ and D_3_ only when S_SLAB_ is the only S allele present. Resistant females from these samples were thus mated with SLAB-TC males (S/S) and their offspring analyzed (see Methods)., Despite the large crossing (700 females from each sample), the number of egg rafts was very low (this is usually observed for the *C. p. pipiens* subspecies, P. Labbé and M. Weill, personal observation), and some females were able to lay eggs several times, so that no reliable frequencies could be estimated. Nevertheless, molecular analyses revealed that both duplications are now segregating in these populations (Table 3).

**Table 3:**
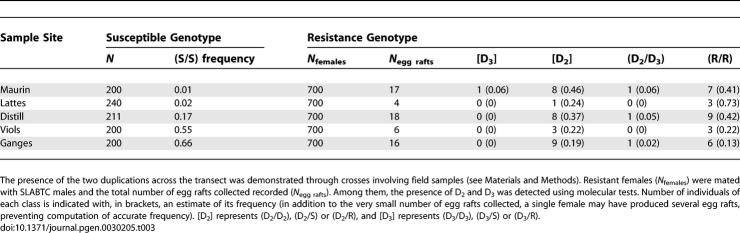
Identifying Duplications in the Field

This plateau frequency pattern strongly suggests that equilibrium has been reached among the four alleles segregating in these populations, S, R, D_2_, and D_3_. The next step in the analysis was therefore to infer, from the clinal patterns observed in the last years (1999–2002), the different genotypic fitnesses (in treated and nontreated areas) that could correspond to this equilibrium. With four alleles, there are ten diploid genotypes and therefore nine relative fitnesses to estimate in each habitat. Since we do not have access to the respective frequencies of D_2_ and D_3_, some of these parameters cannot be estimated separately (see Methods). We report fitness estimates that assume that the two duplications have identical fitness effects. We developed a maximum-likelihood analysis by combining exact simulations with an optimization routine. We used previous estimation for migration: C. pipiens displays a migration of 6.6 km.generation^−1/2^ [26]. This analysis indicated that the stable cline we observe could be expected at equilibrium between migration and selection. To achieve this equilibrium, the different genotypes should display relative fitness, as reported in Table 4. Since the equilibrium situation seems to have been reached around 1995–1996, it therefore suggests that both duplications spread before that date. Importantly, this equilibrium requires a situation of double overdominance: heterozygotes involving duplication, i.e., (D_2_/S), (D_3_/S), (D_2_/D_3_), (D_3_/R), and (D_2_/R), must have a higher fitness than R, D_2_, and D_3_ homozygotes in absence of insecticide. It also requires that R and S homozygotes are the best homozygote genotypes in the treated and nontreated area, respectively. With such a fitness scheme, each duplication could be maintained independently even if they have identical fitness effects. In addition, the presence of both allows them to reach a higher total frequency. More precisely, the overall D frequency when both are present should be intermediate between the frequency they could reach alone and twice this frequency, due to the overdominance of the (D_2_/D_3_) genotype.

**Table 4:**
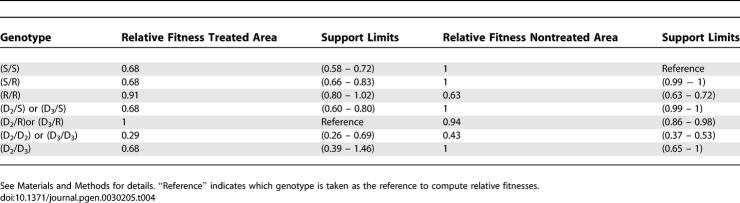
Relative Fitness Estimates Fitted Using Summer Clinal Patterns Observed over the Period 1999–2002

**Figure 3:**
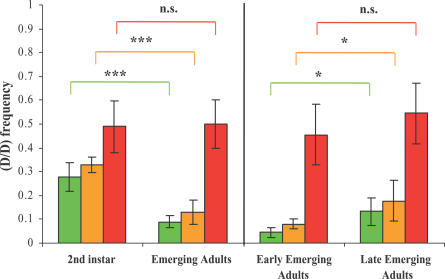
Frequency of Homozygotes (D/D) Compared to Heterozygotes (D/S) during Development The frequency of (D/D) individuals is indicated for second instar and emerging adults on the left. On the right, adults are divided into early and late emerging adults. (i) MAURIN-D ((D_2_/D_2_) versus (D_2_/S), green), (ii) BIFACE-D ((D_3_/D_3_) verus (D_3_/S), orange), and (iii) BIFACE-DFix x MAURIN-D ((D_2_/D_3_) versus (D_3_/S), red). The bars indicate one error-type. Significant frequency differences between the different stages are indicated (n.s., *p*-value > 0.05; *, *p*-value < 0.05; **, *p*-value < 0.01; and ***, *p*-value < 0.001).

**Figure 4:**
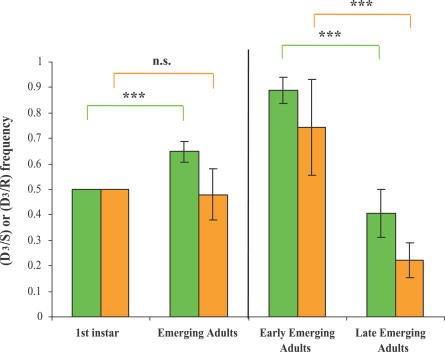
Frequency of Heterozygotes (D/S) and (D/R) Compared to Homozygotes (R/R) during Development The frequency of (D/S) and (D/R) individuals is indicated on the left for two developmental stages, first instar and emerging adults. On the right, emerging adults are divided into early and late emerging adults. (i) (D_3_/S) versus (R/R) (green), (ii) (D_3_/R) versus (R/R) (orange). The bars indicate one error-type. Significant frequency differences between the different stages are indicated (n.s., *p*-value > 0.05; *, *p*-value < 0.05; **, *p*-value < 0.01; and ***, *p*-value < 0.001).

The last step in the analysis was to confirm experimentally this possible pattern of double overdominance. To do so, we followed the relative success, over a single generation in the laboratory, of different pairs of genotypes in the absence of insecticide. In a first set of experiments, we showed that (D_2_/S) and (D_3_/S) largely outperformed (D_2_/D_2_) and (D_3_/D_3_) in terms of survival, development time, and fertility (Figure 3; Tables 5, 6 and 7). Homozygotes for both duplications indeed show an extremely low fitness with high mortality at pupation and emergence along with low fertility, such that it was nearly impossible to fix a strain for these duplications. By contrast, we showed that heterozygotes involving the two duplications (D_2_/D_3_) were as fit as (D_3_/S) (Figure 3; Table 5). Finally, we compared the fitness of (D_3_/S) and (D_3_/R) with R homozygotes (Figure 4; Table 5). Here again, we found that the heterozygotes outperformed the homozygotes, although less strikingly. We were not able to perform this last comparison with the D_2_ duplication because the survival and fertility of (D_2_/D_2_) homozygotes are too low to maintain a laboratory strain fixed for this duplication. Overall, these experiments directly confirm the fitness relationship deduced from the clinal pattern observed in the field. Homozygotes for either D_2_ or D_3_ have a severely reduced fitness, but heterozygotes for the two duplications, (D_2_/S), (D_3_/S), (D_2_/D_3_), and (D_3_/R) perform well.

**Table 5:**
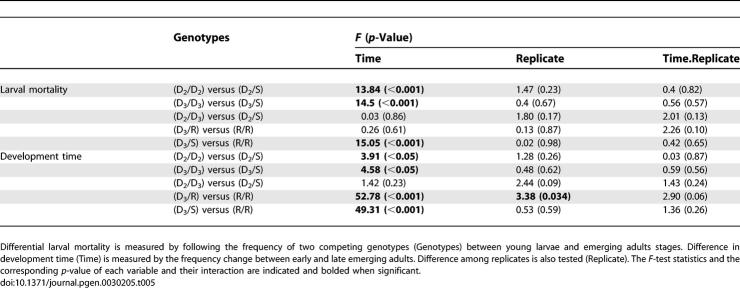
Statistics for Larval Mortality and Development Time

**Table 6:**
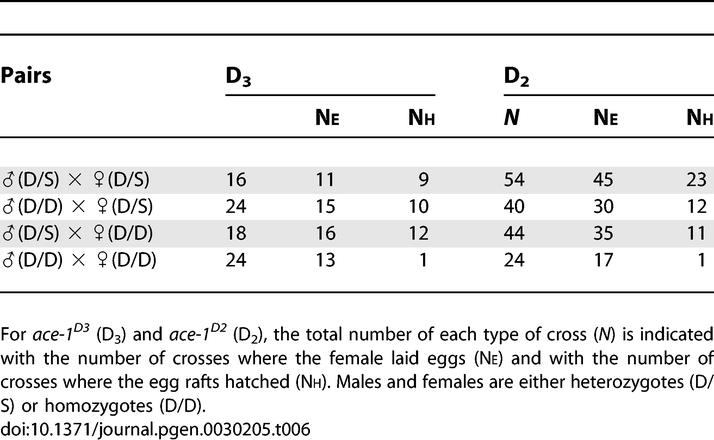
Cross Results for Fertility

**Table 7:**
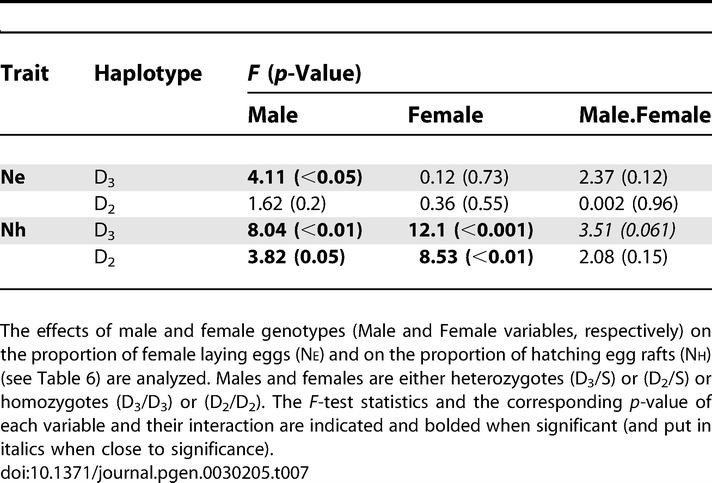
Statistics for Fertility Analysis

## Discussion

This study shows how *ace-1*, one of the two major genes involved in resistance to OP insecticide in the mosquito Culex pipiens, evolved in the last 40 y of control using insecticides. The evolution of resistance to insecticide in C. pipiens does not follow a classical scenario whereby a beneficial mutation with deleterious side effects spreads and is followed by a steady “amelioration” process correcting for these side effects.

About 10 y after the beginning of OP treatments (1977), the major resistance allele *ace-1^R^*, which is beneficial in the treated area but has strong deleterious pleiotropic effects in absence of insecticide, appeared and spread [17,18]. Because of this fitness cost and incoming gene flow from the nontreated area favored by the absence of insecticide treatment in winter, it did not fix but remained polymorphic with a clinal pattern across treated and nontreated areas [26,27,35]. In the early nineties, at least one and probably two duplications involving a resistance and susceptible *ace-1* copy, started to spread and replace *ace-1^R^* [29,30]. Our lab experiments indicate that these two duplications are both severely deleterious when homozygous, but that heterozygotes involving either *ace-1^S^* or *ace-1^R^* alleles do not exhibit deleterious side effects. After ∼1999, the pooled frequency of the duplications does not vary significantly, suggesting that the four “alleles” (*ace-1^S^*, *ace-1^R^*, *ace-1^D2^*, and *ace*-*1^D3^*) reached a stable equilibrium. The stability of the duplicated haplotype frequencies since 1996 suggests that both duplications occurred and spread before 1996. This latter conclusion is tentative, since our data may not be accurate enough to detect the small perturbation of the frequency equilibrium caused by the spread of one of the duplications after 1996, due to the indirect estimation of duplicated haplotype frequency.

Clearly, however, considerable polymorphism in this system is maintained by both overdominance and migration. What is the mechanism by which overdominance operates? First, a duplicated haplotype restores AChE1 activity while maintaining resistance [30]. It therefore combines resistance with no deleterious pleiotropic effect caused by a deficit of AChE1 activity. An excess of AChE1 activity may be deleterious as well, but the one caused by the duplication is mild. For this reason, duplications certainly exhibit at least marginal overdominance over treated and nontreated areas. Duplications could be described as “generalist” haplotypes that perform well in both habitats, compared to the “specialist” alleles *ace*-*1^S^* and *ace-1^R^*. Second, through lab experiments, we found, surprisingly, that both *ace-1^D2^* and *ace-1^D3^* duplications cause very strong deleterious effects when homozygous. However, these effects disappear in *ace-1^D2^*/*ace-1^D3^* heterozygotes. The simplest explanation for this observation is that each duplication occurred independently rather than being generated by recombination (which may not be surprising, given the high duplication rate at this locus [30]), and that they carry distinct recessive sublethal mutations not necessarily related to *ace-1*–mediated resistance. Distinct unequal crossing over could have generated each duplication and in the same time disrupted different genes close to *ace-1*. Alternatively, different sublethal recessive mutations could have hitchhiked with the initial spread of each duplication. In all cases, these explanations require that the duplications cannot get rid of these deleterious mutations by recombining. It therefore suggests that recombination is very low around or within the duplicated haploypes or that they are situated on chromosomal inversions (and thus behave like a “balancer region” in analogy with the balancer chromosomes used in laboratory *Drosophila*). The fact that we never observed in laboratory crosses a recombinant separating the two *ace-1* duplicated copies [30] is consistent with this last hypothesis. We consider this explanation plausible since duplication events involve an important modification of the genome, which can easily disrupt other genes or regulatory regions [31,33,36].

This example of adaptation involves three successful steps (the formation of *ace-1^R^*, *ace-1^D2^* and *ace-1^D3^*), each of them being driven by natural selection. However, each of these three steps presents severe deleterious pleiotropic effects. The deleterious pleiotropic effect of *ace*-*1^R^* may be unavoidable if changing the AChE1 active site to decrease affinity towards OP insecticide necessarily also leads to a lower affinity towards ACh. However, the occurrence and spread of duplications that are sublethal when homozygotic is more perplexing. For instance, another *ace-1* duplication, *ace*-*1^D1^* seems to have spread and be almost fixed on the island of Martinique [37], indicating that *ace-1* duplications do not necessarily involve strong deleterious pleiotropic effects when homozygous (confirmed by laboratory analyses, P. Labbé and M. Weill, unpublished data). However, once a duplication with a recessive cost has spread, even partially, in a population, selection is likely to be less effective at replacing it with a better one because the fate of a beneficial mutation is mostly determined when it is rare and therefore by its heterozygous effect [38]. Since *ace*-*1^D2^* or *ace*-*1^D3^* duplications enjoy almost no fitness cost when heterozygotic, any new duplication has a low chance to spread in these populations (although migration bringing fitter haplotypes could help escaping this apparent dead end). Thus, selection has taken the mosquito populations on a difficult path indeed.

More generally, we may wonder if such a tortuous adaptive trajectory is frequent in nature. Clearly, gene duplications may solve many genetic trade-offs and chromosome rearrangements such as inversions may strongly perturb the genetic architecture. This type of situation may be more common with strong selective pressure and strong pleiotropy, whereas less intense selection may select for more subtle variation (e.g., see [39]). Nevertheless, insecticide resistance may not be an exception, since other selective pressures appear to be intense as well (e.g., parasitism [40,41]) and thus could favor similar complex genetic responses. The new molecular tools available will allow deeper investigation of adaptation genetics evolution, and thus will help to settle the issue of the frequency of the kind of complex patterns uncovered by our study.

In the long term, selection may produce exquisite adaptations, but this study lays bare that even impressive adaptations are likely to have begun with a process of trial and error that seems to be anything but optimal. It appears that natural selection is forced to tinker with available variability, despite the costs, rather than build impressive and cost-free adaptations that are wholly novel.

## Methods

### Data collection.

Data used in cline analysis were collected in the Montpellier area since 1986 along a transect across the treated and the nontreated areas (Figure 2). Published data from the summers of 1986, 1991, 1995, and 1996, spring of 1993, and winters of 1995 and 1996 [26,29,35] were used to perform the overall analysis. This was complemented with unpublished samples: summer of 1999, 2001, and 2002, spring of 1996 and 2000, and winter of 1999 and 2002 (complete dataset in Table S1). For illustration, we also indicate the frequency observed in a single population in 1984 near the coast, but this population was not included in the cline analysis. Five populations were also sampled on the same transect in 2005 (Maurin, Lattes, Distill, Viols, and Ganges) to assess the distribution of the two duplicated haplotypes in the Montpellier area.

### Mosquito strains.

In laboratory experiments, different strains were used to compare life history traits. Two reference strains were used: SLAB, the susceptible reference strain (homozygote for the allele S_Slab_ [34]), and SR, homozygote for *ace-1^R^* (R), but with the same genetic background as SLAB [23]. Two other strains, named MAURIN-D and BIFACE-D and respectively harboring *ace-1^D2^* (D_2_) and *ace-1^D3^* (D_3_), were also used. The duplicated strains originate from MAURIN and BIFACE strains [30] backcrossed for more than 15 generations with SLAB (method in [23]). These strains are not homozygous but contain three genotypes: (S_SLAB_/S_SLAB_), (D/S_SLAB_), and (D/D) (D_2_ and D_3_ for MAURIN-D and BIFACE-D, respectively).

Egg rafts resulting from the cross of homozygous males and females originating from BIFACE-D were used to constitute a strain homozygous for each duplicated haplotype, BIFACE-DFix. SLAB-TC strain (SLAB strain cured from *Wolbachia* bacteria [25]) was used for crosses with field samples to avoid incompatibility phenomena.

### Cline analysis.

*Identification of ace-1 phenotype.* For each mosquito, the head was used to establish the phenotype at the *ace-1* locus, using the TPP test [42], based on enzymatic activity of AChE1 in the presence or absence of insecticide. Single copy allele homozygotes (S/S) and (R/R) are easily detectable using this test. However, the heterozygotes (R/S) and the genotypes involving a duplicated haplotype (heterozygotes: (D/S) and (D/R) and homozygotes: (D/D)) can not be distinguished (they all display a [RS] phenotype). Moreover, this test does not allow distinction between the two duplicated haplotypes D_2_ and D_3_. The pooled frequency of D_2_ and D_3_ can be estimated from the apparent excess of [RS] phenotypes caused by the presence of the duplications [26]. This method assumes Hardy–Weinberg proportions for the different genotypes and is therefore not as accurate as direct observation of the different genotypes. However, it has been shown to correctly estimate D frequency in field samples where duplication frequency was independently estimated using crosses [26]. Note that no molecular test is currently available to directly detect the duplicated haplotypes in natural populations, as their D(R) and D(S) copies are not different from single copy alleles (R and S) present in the same sampling sites.

*Clines description.* We first analyzed the spatial variation in allele frequency across the treated and nontreated areas for each sample independently (i.e., for a given year and season) using a purely descriptive model. We estimated the pooled frequency of both duplications. More specifically, we assumed that the frequency (denoted *p*) of each resistance allele (indicated by the subscript *i* = R or D) at time *j* followed a scaled negative exponential as follows:

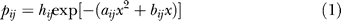
where *x* is the distance from the coast and *h_ij_*, *b_ij_* and *a_ij_* are the estimated parameters. *h_ij_* measures the frequency of resistance allele *i* on the coast (i.e., at *x* = 0) at time *j*. The parameters *b_ij_* and *a_ij_* describe rates of decline of the frequency of allele *i* (at time *j*) with distance and with the square of distance from the coast, respectively. We allow for a flexible clinal shape because it tends to vary with season [35].

The second step in the analysis was to compare frequency patterns across years and seasons. For this purpose, we fitted all samples simultaneously to measure the variation of clines trough time using the method developed in Labbé et al. [16]. More specifically, we assumed that *h_ij_* values changed smoothly as a logistic function of time (measured in months)

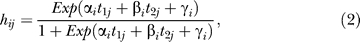
where *t*_1*j*_ is the number of months after January 1986 when the date of sampling is before January 1986 + t^*^ and is t^*^ otherwise. *t*_2*j*_ is the number of months after January 1986 + t^*^. *α_i_*, *β_i_*, *γ_i_*, and t^*^ are estimated parameters. The overall change in frequency over the 1986–2002 period is measured for each resistance allele *i* by *α_i_* and *β_i_*, which measure the rate of frequency change between 1986 and 1986 + t^*^ and between 1986 + t^*^ and 2002, respectively. t^*^ was introduced to allow for changes in the rate of allele replacement. Parameter *γ_i_* is related to the initial frequency *h_i0_* of each allele *i* (*h_i0_* = Exp(*γ_i_*)/[1 + Exp(*γ_i_*)]). Expected phenotypic distributions were computed using allelic frequency and assuming Hardy–Weinberg proportions in each location (see [16]). The phenotype was considered to be a three-state random variable ([RR], [RS], and [SS]). The log-likelihood of a sample was computed from the phenotypic multinomial distribution and maximized using the Metropolis algorithm (see [26,27,35]). Models were compared using *F*-tests in order to correct for overdispersion. Deviance was also corrected for overdispersion to find the support limits of each parameter [43,44].

*Fitness estimation of the different genotypes.* In order to determine the fitness of the different genotypes that would yield the stable clines observed over the period 1999–2002, we also used a maximum-likelihood approach. For a given set of fitness values, we obtained by simulation the distribution of genotypes at equilibrium at different distances from the coast. From this distribution, we computed the expected frequency of [RR], [RS], and [SS] for each population in our dataset. The likelihood was then computed and maximized in the same way as with the descriptive models above. The simulation was performed using a stepping stone across treated and nontreated areas using the dispersion kernel that has been previously estimated for C. pipiens as described in Lenormand et al. [26] but with a treated area of 16 km that reflects the treatment practices over this period (P. Labbé, unpublished data). Because we do not have estimates for the relative frequency of the two duplications, we could not estimate their relative fitness. We therefore report estimates assuming that the two duplications have the same fitness effects. The fitness of a given genotype was modelled with two components: a fitness cost *c* (expressed in both treated and non treated areas) and a reduction in survival due to the presence of insecticide in the treated area *s*. The full parameter range was constrained to reflect that *s* should be lower (or equal) for genotypes with an increasing number *R* copies.

At first it may be surprising that polymorphism with more than two alleles could be maintained at migration—selection equilibrium in a situation with only two habitats (treated and nontreated areas). First, even with haploid selection, the number of alleles that can be maintained is larger than the number of habitats if dispersal is localized (as confirmed by our simulations, see also [45]). Second, particular overdominance relationships among alleles (such as the ones we find) can also increase the number of alleles that can be maintained at equilibrium.

### Distribution of the two duplicated haplotypes.

*Crosses.* Larvae were sampled in five populations along the sampling transect (Maurin, Lattes, Distill, Viols, and Ganges; Figure 2) and reared in the laboratory. They were exposed to a dose of insecticide that kills all [SS] individuals (25 × 10^−6^ M of Propoxur). About 700 resistant females from each population were crossed with about 800 males of SLAB-TC strain (S_SLAB_/S_SLAB_). They were repeatedly blood fed each week until they died. Egg rafts were collected every day, isolated, and reared to the third instar. They were then exposed to a dose of insecticide that kills all [SS] individuals in order to discard all susceptible field alleles resulting from (D/S) or (R/S) mothers. DNA was extracted from a pool of ∼20 survivors of each egg raft. Molecular tests described below were used to detect the presence of each resistance haplotype (R, D_2_, and D_3_). Four types could be identified: genotypes (D_2_/D_3_) and (R/R) and phenotypes [D_2_] (i.e., (D_2_/D_2_), (D_2_/S), or (D_2_/R)), and [D_3_] (i.e., (D_3_/D_3_), (D_3_/S), or (D_3_/R)).

**Molecular tests.**

Using partial *ace-1* sequences of each haplotype [30], we designed RFLP tests to discriminate D_2_, D_3_, R, and S_SLAB_. PCR amplification of a 458 bp fragment of exon 3 using the primers CxEx3dir 5′-CGA CTC GGA CCC ACT CGT-3′ and CpEx3rev 5′-GAC TTG CGA CAC GGT ACT GCA-3′ was performed (30 cycles, 93 °C for 30s, 55 °C for 30s, and 72 °C for 1min). The amplified fragment was then digested in parallel by different restriction enzymes. First, the fragment was cut twice by the enzyme BsrBI only when S_SLAB_ is present, generating three fragments (127 bp, 141 bp, and 190 bp; Figures S1 and S2), all the other alleles being cut only once (two fragments of 127 bp and 331 bp; Figures S1 and S2). Second, the fragment is cut by the enzyme EagI only when D_2_(S) is present, generating two fragments (150 bp and 308 bp; Figures S1 and S2). Third, the fragment is cut by the enzyme HinfI only when D_3_(S) is present, generating two fragments (102 bp and 354 bp; Figures S1 and S2; note that there is a HinfI site in the primer CxEx3dir, subtracting 2 bp in each fragment; Figure S1). Discriminating between the resistance and susceptible copies is possible using the test provided by Weill et al. [20]: the G119S mutation providing resistance creates a site for the enzyme AluI.

### Laboratory experiments.

Larval mortality: *(D/D) versus (D/S).* Trials between (D/D) and (D/S) individuals were performed in triplicate. Larvae of different genotypes were reared in competition under the same environmental conditions (food, temperature, etc.). They were selected at the first instar stage using Propoxur at a concentration of 25 × 10^−6^ M, which eliminates only (S/S) individuals. Adults were collected during the first and the second wk after the first adult emergence (∼30 individuals each wk). They will be respectively referred as early (first wk) and late (second wk) emerging adults. Genotype frequency was measured at second larvae instar and both adulthood stages. Three trials were conducted: (i) (D_3_/S) versus (D_3_/D_3_), (ii) (D_2_/S) versus (D_2_/D_2_), and (iii) (D_3_/S) versus (D_2_/D_3_). The different genotypes were obtained respectively from (i) a cross between males and females from BIFACE-D (progeny genotypes (D_3_/D_3_), (D_3_/S_SLAB_), and (S_SLAB_/S_SLAB_)), (ii) a cross between males and females from MAURIN-D (progeny genotypes (D_2_/D_2_), (D_2_/S_SLAB_), and (S_SLAB_/S_SLAB_)) and (iii) a cross between males from BIFACE-DFix and females from MAURIN-D and the reverse cross (progeny genotypes (D_2_/D_3_) and (D_3_/S_SLAB_)). Each sample was analyzed using the BsrBI-based RFLP test to determine the proportion of individuals of genotype (D_3_/S_SLAB_) (in the first and third trials) or (D_2_/S_SLAB_) (in the second trial).

*Larval mortality: (D/S) or (D/R) versus (R/R)*. Trials were conducted between (D/R) or (D/S) individuals and individuals homozygote for the single resistance allele (R/R). Trials were performed in triplicates with 500 first instar larvae of each genotype reared under the same environmental conditions (food, temperature, etc.). In each replicate, early and late emerging adults were collected as indicated above. Two trials were carried out: (i) (D_3_/R) versus (R/R) and (ii) (D_3_/S) versus (R/R). The different genotypes were obtained from (i) a cross between females from BIFACE-DFix and males from strain SR to obtain (D_3_/R) individuals, and (ii) a cross between females from BIFACE-DFix and males from SLAB to obtain (D_3_/S_SLAB_) individuals. (R/R) individuals were directly obtained from strain SR. Each sample was analyzed using the AluI-based RFLP test to determine the proportion of individuals of genotype (D_3_/R) (first trial) or (D_3_/S_SLAB_) (second trial).

*Fertility*. Different crosses were realized in order to determine the fertility of individuals carrying a duplicated allele at the homozygous or heterozygous state ((D/D) or (D/S)). In each case, the proportion of females laying eggs and the proportion of hatching eggs rafts was recorded. For each series of cross, about 50 males were mated independently with five females each, all from the same strain. Five days after mating, the genotype of the males was determined using the BsrBI-based RFLP test. Females were then grouped according to the male genotype, blood fed, and kept without access to laying substrate. Six days later, they were allowed to lay eggs individually. The females were genotyped either after they laid eggs or less than 6 h after their death. Two series of crosses were realized (i) between males and females from BIFACE-D (genotype (D_3_/D_3_) or (D_3_/S_SLAB_) after selection), and (ii) between males and females from MAURIN-D (genotype (D_2_/D_2_) or (D_2_/S_SLAB_) after selection), to assess the fertility of individuals carrying *ace-1^D3^* and *ace-1^D2^*, respectively.

Statistical analysis. For the larval mortality analysis, the following generalized linear model (GLM) was fitted, with binomial error: DD = Time + Replicate + Time.Replicate, where DD represents the proportion of the (D/D) genotype in the population (i.e., (D_3_/D_3_), (D_2_/D_2_), and (D_2_/D_3_) for the first, second, and third crosses, respectively), Time is a factor indicating when the sample was taken, Replicate is a factor indicating the three containers in which the experiment was replicated, and Time.Replicate is the interaction between the two factors. Two analyses were performed: (i) one to test for a difference in mortality between (D/D) and (D/S) individuals (in that case, Time was 2nd instar or emerging adult), and (ii) the second to test for a difference in development time between (D/D) and (D/S) individuals (in that case, Time was early emerging or late emerging adults). A similar model was used to analyze the proportion of (D_3_/S) and (D_3_/R) in trials versus (R/R). These models were simplified according to Crawley [46]: significance of the different terms was tested starting from the higher-order terms using *F*-test. Nonsignificant terms (*p* > 0.05) were removed. Factor levels of qualitative variables that were not different in their estimates (using *F*-test) were grouped as described by Crawley [46]. This process yielded a minimal adequate model.

The fertility of males and females of different genotypes was analyzed by comparing the proportion of females laying eggs and the proportion of hatching egg rafts among the different types of cross. The number of females laying eggs (Ne) and the number of hatching egg rafts (Nh) were analyzed using GLM with binomial error: Male + Female + Male.Female, where Male and Female are factors indicating male (or female) genotype. These models were simplified as above. All analyses were performed using R software (v 2.0.1., http://www.r-project.org).

## Supporting Information

Figure S1 Susceptible and Resistance Copy Sequences The sequence of R, S_SLAB_, D_2_(S), and D_3_(S) are presented. The restriction enzyme sites specific to each copy are also indicated (AluI [blue], BsrBI [yellow], HinfI [green], and EagI [red] for R, S_SLAB_, D_2_(S), and D_3_(S), respectively; see Materials and Methods and Figure S2].
(34 KB DOC)

Figure S2 RFLP Profiles The RFLP profiles allowing the identification of the different genotypes are presented for homozygotes S_SLAB,_ R, D_2_, and D_3_. For each genotype, three restriction enzymes are used (BsrBI, HinfI, and EagI; Figure S1) on the CxEx3dir-CpEx3rev PCR product (see Methods) and an undigested control is also presented. Note that for D_2_ and D_3_ homozygotes, both the susceptible and the resistant copies are present in the PCR product, thus leading to an apparent heterozygote (e.g., for the (D_2_/D_2_) genotype only the D_2_(S) copy is digested by EagI, not the D_2_(R) copy).
(285 KB DOC)

Table S1 Sampling Data For each year, the numbers of individuals carrying each phenotype are indicated: (A) in spring, (B) in summer, and (C) in winter. For nomenclature, [SS] and [RR] phenotypes stand for (S/S) and (R/R) genotypes, whereas [RS] phenotype covers all (R/S), (D/S), (D/R), and (D/D) genotypes. (−) indicates that the site was not sampled in the corresponding year. See Figure 2 for details of sampling location.
(47 KB DOC)

### Accession Numbers

The National Center for Biotechnology Information (NCBI) GenBank database (http://www.ncbi.nlm.nih.gov/sites/entrez?db=Nucleotide) accession numbers for *ace-1* are AJ489456 and AJ515147.

## Abbreviations

AChE1: acetylcholinesterase
OP: organophosphorous

## References

[pgen-0030205-b001] Fisher RA (1930). The genetical theory of natural selection.

[pgen-0030205-b002] Hartl D, Taubes C (1996). Compensatory nearly neutral mutations: selection without adaptation. J Theor Biol.

[pgen-0030205-b003] Duboule D, Wilkins AS (1998). The evolution of ‘bricolage'. Trends Genet.

[pgen-0030205-b004] Jacob F (1977). Evolution and Tinkering. Science.

[pgen-0030205-b005] Caspari E (1952). Pleiotropic gene action. Evolution.

[pgen-0030205-b006] Lande R (1983). The response to selection on major and minor mutations affecting a metrical trait. Heredity.

[pgen-0030205-b007] Wright S (1969). Evolution and the genetics of populations.

[pgen-0030205-b008] Carrière Y, Deland J-P, Roff DA, Vincent C (1994). Life-history cost associated with the evolution of insecticide resistance. Proc R Soc Lond B Biol Sci.

[pgen-0030205-b009] Otto SP (2004). Two steps forward, one step back:the pleiotropic effects of favoured alleles. Proc R Soc Lond B Biol Sci.

[pgen-0030205-b010] Cohan FM, King EC, Zawadzki P (1994). Amelioration of the deleterious pleiotropic effects of an adaptive mutation in Bacillus subtilis. Evolution.

[pgen-0030205-b011] Fisher RA (1928). The possible modification of the response of the wild type to recurrent mutations. Am Nat.

[pgen-0030205-b012] Haldane JBS (1932). The Causes of Evolution.

[pgen-0030205-b013] Lenski RE (1988). Experimental studies of pleiotropy and epistasis in *Escherichia coli. II.* Compensation for maladaptive effects associated with resistance to virus T4. Evolution.

[pgen-0030205-b014] McKenzie JA (1996). Ecological and evolutionnary aspects of insecticide resistance.

[pgen-0030205-b015] Guillemaud T, Lenormand T, Bourguet D, Chevillon C, Pasteur N (1998). Evolution of resistance in Culex pipiens: allele replacement and changing environment. Evolution.

[pgen-0030205-b016] Labbé P, Lenormand T, Raymond M (2005). On the worldwide spread of an insecticide resistance gene: a role for local selection. J Evol Biol.

[pgen-0030205-b017] Raymond M, Berticat C, Weill M, Pasteur N, Chevillon C (2001). Insecticide resistance in the mosquito Culex pipiens: what have we learned about adaptation?. Genetica.

[pgen-0030205-b018] Weill M, Labbé P, Duron O, Pasteur N, Fort P, Fellowes MDE, Holloway GJ, Rolff J (2005). Insecticide resistance in the mosquito Culex pipiens: towards an understanding of the evolution of *ace* genes. Insect evolutionary ecology.

[pgen-0030205-b019] Weill M, Lutfalla G, Mogensen K, Chandre F, Berthomieu A (2003). Insecticide resistance in mosquito vectors. Nature.

[pgen-0030205-b020] Weill M, Malcolm C, Chandre F, Mogensen K, Berthomieu A (2004). The unique mutation in *ace-1* giving high insecticide resistance is easily detectable in mosquito vectors. Insect Mol Biol.

[pgen-0030205-b021] Alout H, Berthomieu A, Cui F, Tan Y, Berticat C (2007). Different amino-acid substitutions confer insecticide resistance through acetylcholinesterase 1 insensitivity in Culex vishnui and Culex tritaeniorhynchus (Diptera: Culicidae) mosquitoes from China. J Med Entomol.

[pgen-0030205-b022] Bourguet D, Lenormand T, Guillemaud T, Marcel V, Fournier D (1997). Variation of dominance of newly arisen adaptative genes. Genetics.

[pgen-0030205-b023] Berticat C, Boquien G, Raymond M, Chevillon C (2002). Insecticide resistance genes induce a mating competition cost in Culex pipiens mosquitoes. Genet Res.

[pgen-0030205-b024] Bourguet D, Guillemaud T, Chevillon C, Raymond M (2004). Fitness costs of insecticide resistance in natural breeding sites of the mosquito Culex pipiens. Evolution.

[pgen-0030205-b025] Duron O, Labbe P, Berticat C, Rousset F, Guillot S (2006). High *Wolbachia* density correlates with cost of infection for insecticide resistant Culex pipiens mosquitoes. Evolution.

[pgen-0030205-b026] Lenormand T, Guillemaud T, Bourguet D, Raymond M (1998). Evaluating gene flow using selected markers: a case study. Genetics.

[pgen-0030205-b027] Lenormand T, Bourguet D, Guillemaud T, Raymond M (1999). Tracking the evolution of insecticide resistance in the mosquito Culex pipiens. Nature.

[pgen-0030205-b028] Bourguet D, Raymond M, Bisset J, Pasteur N, Arpagaus M (1996). Duplication of the *Ace.1* locus in Culex pipiens from the Caribbean. Biochem Genet.

[pgen-0030205-b029] Lenormand T, Guillemaud T, Bourguet D, Raymond M (1998). Appearance and sweep of a gene duplication: adaptive response and potential for new functions in the mosquito Culex pipiens. Evolution.

[pgen-0030205-b030] Labbé P, Berthomieu A, Berticat C, Alout H, Raymond M (2007). Independent duplications of the acetylcholinesterase gene conferring insecticide resistance in the mosquito Culex pipiens. Mol Biol Evol.

[pgen-0030205-b031] Ohno S (1970). Evolution by gene duplication.

[pgen-0030205-b032] Lynch M, Conery JS (2000). The evolutionary fate and consequences of duplicate genes. Science.

[pgen-0030205-b033] Otto S, Yong P (2002). The evolution of gene duplicates. Adv Genet.

[pgen-0030205-b034] Georghiou GP, Metcalf RL, Gidden FE (1966). Carbamate-resistance in mosquitoes: selection of *Culex pipiens fatigans* Wied (= Culex quinquefasciatus) for resistance to Baygon. Bull World Health Organ.

[pgen-0030205-b035] Lenormand T, Raymond M (2000). Analysis of clines with variable selection and variable migration. Am Nat.

[pgen-0030205-b036] Kirkpatrick M, Barton N (2006). Chromosome inversions, local adaptation and speciation. Genetics.

[pgen-0030205-b037] Yebakima A, Marquine M, Rosine J, Yp-Tcha MM, Pasteur N (2004). Evolution of resistance under insecticide selection pressure in *Culex pipiens quinquefasciatus* (Diptera, Culicidae) from martinique. J Med Entomo.

[pgen-0030205-b038] Roze D, Rousset F (2003). Selection and drift in subdivided populations: a straightforward method for deriving diffusion approximations and applications involving dominance, selfing and local extinctions. Genetics.

[pgen-0030205-b039] McKenzie JA, Parker AG, Yen JL (1992). Polygenic and single gene responses to selection for resistance to diazinon in Lucilia cuprina. Genetics.

[pgen-0030205-b040] Little TJ, Watt K, Ebert D (2006). Parasite-host specificity: experimental studies on the basis of parasite adaptation. Evolution.

[pgen-0030205-b041] Carton Y, Nappi AJ, Poirie M (2005). Genetics of anti-parasite resistance in invertebrates. Dev Comp Immunol.

[pgen-0030205-b042] Bourguet D, Pasteur N, Bisset J, Raymond M (1996). Determination of Ace.1 genotypes in single mosquitoes: toward an ecumenical biochemical test. Pestic Biochem Physiol.

[pgen-0030205-b043] Lebreton J-D, Burnham KP, Clobert J, Anderson DR (1992). Modeling survival and testing biological hypotheses using marked animals: a unified approach with case studies. Ecol Monogr.

[pgen-0030205-b044] Anderson DR, Burnham KP, White GC (1994). AIC model selection in overdispersed capture-recapture data. Ecology.

[pgen-0030205-b045] Nagylaki T, Lou Y (2001). Patterns of multiallelic polymorphism maintained by migration and selection. Theor Popul Biol.

[pgen-0030205-b046] Crawley MJ (2003). Statistical computing. An introduction to data analysis using S-plus.

