# Supplementary material for: Forty Years of Erratic Insecticide Resistance Evolution in the Mosquito Culex pipiens
Source: PLoS Genet. 2007 Nov 16;3(11):e205. doi: 10.1371/journal.pgen.0030205 (PMC2077897; doi:10.1371/journal.pgen.0030205)
Supplement: Supplementary file 1 [file pgen.0030205.sg001.doc]

1	70

S_SLAB_	CGACTCGGAC CCACTCGTCA TAACGACGGA CAAGGGCAAA ATCCGTGGAA CGACACTGGA AGCGCCTAGT

R	---------- --------A- ---------- ---------- ---------- ---------- ------A---

D_2_(S)	---T------ --------A- ---------- ---------- ---------- ---------- ------A---

D_3_(S)	---------- --------A- ---------- ---------- ---------- ---------- ------A---

```
            HinfI  CxEx3dir
            71                                                                       140
```

S_SLAB_	GGAAAGAAGG TGGACGCATG GATGGGCATT CCGTACGCGC AGCCCCCGCT GGGTCCGCTC CGGTTTCGAC

R	---------- ---------- ---------- ---------- ---------- ---------- ----------

D_2_(S)	-----A---- ---------- ---------- ---------- ---------- ---------- ----------

D_3_(S)	---------- ---------- ---------- ---------- ---------- ---------- ----------

```
								 	    BsrBI
            141                                                                      210
```

S_SLAB_	ATCCGCGACC CGCCGAAAGA TGGACCGGTG TGCTGAACGC GACCAAACCG CCCAACTCCT GCGTCCAGAT

R	---------- ---------- ---------- ---------- ---------A ---------- ----------

D_2_(S)	-------T-- ---------- ---------- ---------- ---------G ---------- ----------

D_3_(S)	---------- ---------- ---------- ---------- ---------C ---------- ----------

211	280

S_SLAB_	CGTGGACACC GTGTTCGGTG ACTTCCCGGG GGCCACCATG TGGAACCCGA ACACACCGCT CTCGGAGGAC

R	---------- ---------- ---------- ---------- ---------- -------C-- ----------

D_2_(S)	---------- ---------- ---------- ---------- ---------- -------C-- ----------

D_3_(S)	---------- ---------- ---------- ---------- ---------- -------C-- ----------	**BsrBI**

281	350

S_SLAB_	TGTCTGTACA TCAACGTGGT CGTGCCACGG CCCAGGCCCA AGAATGCCGC CGTCATGCTG TGGATCTTCG

R	---------- ---------- -------A-- --G------- ---------- T--------- --------T-

D_2_(S)	---------- ---------- ---------- --G------- ---------- ---------- --------T-

D_3_(S)	---------- ---------- ---------- ---------- ---------- ---------- --------T-

```
					    EagI
            351                                                                      420
```

S_SLAB_	GGGGTGGCTT CTACTCCGGG ACTGCCACGC TGGACGTGTA CGACCACCGG ACGCTGGCCT CGGAGGAGAA

R	-----A---- ---------- ---------T ---------- ---T—-T--- ---------- ----------

D_2_(S)	---------- ---------- ---------T ---------- ------T--- ---------- ----------

D_3_(S)	-------A-- ---------- ---------T ---------- ---------- ---------- ----------

```
	        AluI HinfI
            421                                   458
```

S_SLAB_	CGTGATCGTA GTTTCGCTGC AGTACCGTGT CGCAAGTC

R	---------G ---------- ---------- --------

D_2_(S)	---------G ---------- ---------- --------

D_3_(S)	T--------G ---------- ---------- --------

CpEx3rev
