# Supplementary material for: Forty Years of Erratic Insecticide Resistance Evolution in the Mosquito Culex pipiens
Source: PLoS Genet. 2007 Nov 16;3(11):e205. doi: 10.1371/journal.pgen.0030205 (PMC2077897; doi:10.1371/journal.pgen.0030205)
Supplement: Supplementary file 3 [file pgen.0030205.st001.doc]

**a)**

**b)**

**c)**
